# Polymicrobial infections and microbial patterns in infected nonunions – a descriptive analysis of 42 cases

**DOI:** 10.1186/s12879-020-05386-9

**Published:** 2020-09-10

**Authors:** Markus Rupp, Stefanie Kern, Tobias Weber, Tamina D. Menges, Reinhard Schnettler, Christian Heiß, Volker Alt

**Affiliations:** 1 Department for Trauma, Hand and Reconstructive Surgery, University Hospital Giessen and Marburg, Campus Giessen, Rudolf-Buchheim-Straße 7, 35385 Giessen, Germany; 2 Department for Trauma Surgery, University Medical Center Regensburg, Franz-Josef-Strauß-Allee 11, 93053 Regensburg, Germany; 3 Experimental Trauma Surgery, Justus-Liebig-University Giessen, Aulweg 128, 35385 Giessen, Germany; 4 Faculty of Medicine, Justus-Liebig-University Giessen, Klinikstraße 29, 35385 Giessen, Germany

**Keywords:** Nonunion, Pseudarthrosis, Bone infection, Germ, Pathogen, *Staphylococcus aureus*, Polymicrobial infection

## Abstract

**Background:**

Frequencies of polymicrobial infection and pathogens evidenced in course of infected nonunion treatment are largely unknown. Therefore, this study aims at investigating microbial patterns in infected nonunions.

**Methods:**

Surgically treated patients with long bone infected nonunion admitted between January 2010 and March 2018 were included in the study. Microbiological culture and polymerase-chain-reaction results of tissue samples of initial and follow-up revision surgeries were assessed and compared with patient and treatment characteristics.

**Results:**

Forty two patients with a mean age of 53.9 ± 17.7 years were included. In six patients (14.3%) polymicrobial infection was evident. A change of pathogens evidenced in course of the treatment occurred in 21 patients (50%). In 16 patients (38.1%) previously detected bacteria could be determined by microbial testing after further revision surgery. *Staphylococcus aureus* was most often detected (*n* = 34, 30.6%), followed by *Enterococcu*s spp. (*n* = 25, 22.5%) and *Staphylococcus epidermidis* (*n* = 18, 16.2%). Five *Staphylococcus aureus* were resistant to methicillin (MRSA). In patients without polymicrobial infection or further germ detection in course of the treatment, 86.4% of the infections were due to *Staphylococcus* spp.. Infections due to *Streptococcus* spp. and gram-negative bacteria were only present in patients with polymicrobial infection and germ-change in course of the treatment.

**Conclusion:**

A low rate of polymicrobial infections was evidenced in the present study. Germ-change often occurs in course of revision surgeries. Prospective studies with more sensitive diagnostic tools are necessary to elucidate the therapeutical relevance of microbiological testing results for surgical as well as medical treatment in infected nonunions.

## Background

Nonunions remain one of the most devasting problems in orthopedic surgery. While overall nonunion rates are reported to range from 1.9–10%, treatment lasts often several months if not years [1]. Besides significant treatment costs [2], the burden for each patient suffering from nonunion is high. Lower leg limb nonunions are reported to come along with significant physical and mental disabilities [3]. If nonunion appears, an infection leading to disturbed bone healing should always be considered possible. Similar to periprosthetic joint infections (PJI), criteria for fracture related infections have been defined. Besides clinical signs such as fistula and pus during surgery, microbiological determination of pathogens by culture from at least two tissue specimens and histopathological evidence of pathogens are deemed to confirm infection [4]. For diagnosis as well as treatment, proof of the infection causing pathogen is regarded being crucial. Treatment is usually performed in a staged-manner by infect eradication and bone reconstruction at a later time point [5]. The use of local antibiotics most commonly incorporated into polymethylmethacrylate (PMMA) cement in different forms (e.g. spacers, beads, coated rods/wires) and additional systemic antibiotics remain a mainstay of this treatment concept [6]. While polymicrobial infections in PJI are expected to complicate treatment and outcome [7, 8], pathogen patterns in diagnosing infected nonunion have not been investigated yet. Several factors such as prior fracture treatment with prophylactic and in case of open fractures often prolonged use of broad-spectrum antibiotics might influence the germ spectrum and occurrence of polymicrobial infections in infected nonunions. Therefore this study aims to investigate the frequency of polymicrobial infection and pathogen types in patients treated for infected nonunions.

## Methods

The local institutional review board “ethics committee of the Justus-Liebig-University Giessen” approved the study beforehand, AZ 68/18. Informed consent from the patients for retrospective analysis of medical records was not necessary. Data was obtained of the clinical electronical data processing system. We retrospectively reviewed medical records of all surgically treated patients suffering from infected nonunions between January 2010 and March 2018. Nonunions of the long bones of the arms and legs (femur, tibia, fibula, humerus, radius and ulna) were included. Patients enrolled in the study had to be 18 years or older at time of revision surgery.

### Fractures and nonunions

Nonunion was defined by failure of fracture healing for at least six months. Patients with delay in fracture healing less than six months were excluded from the study. According to the fracture related infection criteria previously defined by Metsemakers and coworkers [4] infected nonunion was diagnosed if one or more of the following criteria were present: presence of a sinus tract, purulent discharge, exposed osteosynthesis material, positive “probe to implant” test, histologically confirmed infection (> 5 granulocytes per field of view at a at a magnification of 400), > 2000 leucocytes/μl in synovial fluid or > 70% granulocytes of cells in synovial fluid of concomitant infected arthritis. For analyzing microbiological patterns, in all included patients germ detection in microbiological analysis was mandatory. Suggestive parameters such as patients’ medical history and symptoms like erythema, swelling, rest pain and pain on weight bearing, elevated infection parameters in laboratory tests (white blood cell count, C-reactive protein) and radiological signs of infection (osteolysis, implant loosening, sequester formation) were not used for diagnosing infected nonunion.

Fractures leading to nonunion were classified in accordance to the AO/OTA fracture classification. Besides radiological fracture classification, fractures were classified as closed and open fractures.

### Microbiological diagnosis

Microbiological cultures of intraoperatively taken tissue samples were created after each revision surgery. In general, during surgical routine at least three tissue samples were taken for microbiological results. These samples were disrupted using glass beads in tissue rupture device. The homogenate was plated out on solid agar plates such as Brain heart infusion (BHI) agar, Columbia agar and MacConkey agar plates at 37 °C. A prolonged incubation time of 14 days was generally performed to improve the sensitivity of diagnostic yield especially of low-virulent and slow growing microorganisms (i.e. Cutibacterium acnes). The identification of the bacteria was performed using Vitek 2 system (BioMérieux, Marcy L’Etoile, France).

Sonication was introduced in our laboratory in 2016. For sonication the implants were covered with either Ringer’s solution or physiological saline solution. The implants were shaken vigorously (30 s) and for 1 min exposed to ultrasound (40 kHz, 0.1–1 W/cm2). The resulting sonication fluid was microbiologically processed and the amount of bacteria was quantitatively assessed (number of colony-forming units per millilitre of sonication fluid) as mentioned before [9]. The detection of ≥50 colony forming units (CFU)/ml was deemed a strong indicator for an implant-associated infection, since only multilayer biofilms can lead to this high bacterial density [10].

In cases with negative culture results, 16S RNA polymerase chain reaction (PCR) was generally performed. For 16S rRNA PCR, DNA was extracted from 200 mg of tissue or 400 ml of synovial fluid using the QIAmp DNA minikit method (Qiagen, Hilden Germany) according to the manufacturer’s protocol. 16S rRNA was amplified using the universal primers 536F (5′-CAGCAGCCGCGGTAATAC-3′) and rp2 (5′-ACGGCTACCTTGTTACGACTT-3′). These PCR products are sequenced both sides and detection of the bacteria was performed by comparing of sequences with GenBank database using BLAST program available at the National Center for Biotechnology Information (NCBI) [11]. Antimicrobial susceptibility was tested using standard microbiological techniques. Mono- and polymicrobial infections as well as type of pathogens were determined. Pathogens detected in course of revision surgeries for infected nonunion treatment were registered.

### Data collection

#### Patients and treatment

Patients’ medical records were searched for patient specific characteristics such as gender, age, body-mass-index (BMI) and American Society of Anaesthesiologists (ASA) classification. In addition, initial trauma mechanism, duration and type of preemptive antibiotic treatment, number of surgeries prior to initial nonunion surgery were assessed as well as as former treatment at referral hospitals. The period from fracture to nonunion revision surgery was determined. We searched whether metal implants were in situ at revision surgery.

### Statistical analysis

Data were analyzed using SPSS statistics version 24.0 (IBM, SPSS Inc., Armonk, NY). Frequencies for all nonunion risk factors were calculated. For analyses of differences between patients, the chi-squared test or Fischer’s exact test was applied for categorical variables. Mann-Whitney U-tests were applied for between-group comparisons. A *p*-value less than 0.05 was considered significant.

## Results

### Demographic data

In total, 42 patients were diagnosed with infected nonunion. Mean age was 53.9 ± 17.7 years (range, 23–95). Overall, 25 (59.5%) of the patients were male and 17 (40.5%) were female. Demographics of patients showed that infected nonunions occurred most often in patients with fractures at the tibia/fibula (59.5%) compared with other fracture sites (humerus, radius/ulna, femur). Open fractures led to infected nonunion in 18 of 42 cases (42.9%). A BMI > 30 kg/m^2^ was evidenced in 17 (42.5%) of all patients. Most patients with infected nonunions were classified as ASA class II (*n* = 26, 61.9%) (Table 1).

**Table 1:** The table shows numbers of infected nonunion differentiated in type of infection (monomicrobial vs. polymicrobial) and cases in which germ-change occurred in course of surgical treatment. Those data are compared for different demographic data as well as fracture type (closed to open fractures). No significant difference was evidenced comparing infection types and germ change for demographic data and fracture type except previous revision surgery at a referral hospital

		infected nonunion	Infection Type			
		[n / %]	Mono [n / %]	Poly [n / %]	Germ Change [n / %]	Pearson χ^**2**^ Test [p-value]
**patients**	total number	42 / 100	21 / 50.0	6 / 14.3	15 / 35.7	–
**gender**	female	17 / 40.5	10 / 47.6	4 / 66.7	3 / 20.0	0.13
	male	25 / 59.5	11 / 52.4	2 /33.3	12 / 80.0	
**fracture localization**	humerus	2 / 4.8	1 / 4.8	0 / 0	1 /6.7	
	radius/ulna	3 / 7.1	0 / 0	0 / 0	3 / 20.0	
	femur	12 / 28.6	5 / 23.8	3 / 50.0	4 / 26.7	0.24
	tibia/fibula	25 / 59.5	15 / 71.4	3 / 50.0	7 / 46.7	
**ASA score**	1	2 / 4.8	0 / 0	1/ 20.0	1 / 7.1	
	2	26 / 61.9	12 / 57.1	2 / 40.0	12 / 85.7	0.05
	3	12 / 28.6	9 / 42.9	2 / 40.0	1 / 7.1	
**BMI [kg/m**^**2**^**]**	< 18.5	–				0.51
	18.5–25.0	16 / 39.0	8 / 38.1	3 / 60.0	5 / 33.3	
	25.0–30.0	7 / 17.1	2 / 9.5	1 /20.0	4 / 26.7	
	> 30.0	18 / 43.9	11 / 52.4	1 / 20.0	6 / 43.9	
**fracture**	closed	24 / 57.1	11 / 52.4	3 / 50.0	10 /66.7	0.69
	open	18 / 42.9	10 / 47.6	3 / 50.0	5 / 33.3	
**revision surgery at referral hospital**	yes	26 / 61.9	16 / 76.2	5 / 83.3	5 / 33.3%	0.01
	no	16 / 38.1	5 / 23.8	1 / 16.7	10 / 66.7%	

### Monomicrobial and polymicrobial infections

Polymicrobial infections could be evidenced in 6 patients (14.3%). No difference between monomicrobial and polymicrobial infections was found comparing gender, fracture localization, BMI and ASA score. Intriguingly, no difference between closed and open fractures could be determined as well. Twenty one patients (50%) suffered from monomicrobial infected nonunion. In 15 patients an additional germ-change in course of the treatment was evidenced (35.7%). Eleven patients (52.4%) who initially suffered a closed fracture were treated due to monomicrobial infected nonunion. Ten patients (47.6%) were treated for monomicrobial infected nonunion after open fracture. Of the 6 cases in which polymicrobial infection was evidenced three cases were initially open and closed fractures, respectively. Germ-changes were detected in ten cases after closed fracture (66.7%). In five cases (33.3%) patients had an open fracture which led to infected nonunion. No significant difference between open and closed fractures was determined in respect to polymicrobial infection as well as germ-change. Germ-changes occurred all (21/21 patients, 100%) in patients with initial monomicrobial infection. Similar to this observation repeated detection of the same pathogen was observed in 15 out of 16 patients (93.8%) (Table 2). Twenty-six patients have been treated surgically for infected nonunion at referral hospitals prior to admission at our hospital (26/42, 61.9%) (Table 1). Five of six patients with polymicrobial infections evidenced after initial revision surgery have been treated surgically before at referral hospitals due to infected nonunion (5/6, 83.3%). Regarding role of trauma and aspects of initial treatment such as type of antibiotic treatment, duration of antibiotic treatment and remaining metal in situ at nonunion revison surgery, no significant difference between monomicrobial and polymicrobial nonunions could be evidenced. In addition, no difference was detected when cases with germ-changes were compared to both other groups (Table 3).

**Table 2:** Both repeated germ detection and detection of different germs in course of follow-up revision surgeries were significantly more evident in cases with monomicrobial infection at initial revision surgery, *p* < 0.05

type of infection		(n / %)
repeated detection of the same pathogen in course of follow-up surgeries	total	16 / 100
	monomicrobial	15 / 93.8
	polymicrobial	1 / 6.2
germ changes in course of follow-up surgeries	total	21 / 100
	monomicrobial	21 / 100
	polymicrobial	0

**Table 3:** No difference regarding infection type (monomicrobial, polymicrobial, germ-change) was evident when compared trauma/accident, antibiotics initially used and duration of initial antibiotic treatment. The same applies for metallic implants which still were in place at time of revision surgery and previous revision surgeries for infected nonunion at referral hospitals prior to definitive surgical treatment

		infection type			Pearson χ^2^ Test	
		mono [n / %]	poly [n / %]	germ-change [n / %]	[value / df]	p-value
trauma / accident	pathological	0 / 0	0 / 0	1 / 7.1	13.486 / 16	0.701
	motorcycle	1 / 4.5	0 / 0	1 /7.1		
	car accident	5 / 22.7	1 / 16.7	1 / 7.1		
	fall > 3 m	2 / 9.1	0 / 0	1 / 7.1		
	fall < 3 m	7 / 31.8	3 / 50.0	5 / 35.7		
	pedestrian	2 / 9.1	2 / 33.3	2 / 14.3		
	shot	2 / 9.1	0 / 0	0 / 0		
	unknown	1 / 4.5	0 / 0	3 / 21.4		
	bicycle	2 / 9.1	0 / 0	0 / 0		
antibiotics	unknown	9 / 40.9	0 / 0	4 / 26.7	11.459 / 10	0.307
	cefazolin	8 / 36.4	5 / 83.3	7 / 46.7		
	ciprofloxacin	1 / 4.5	0 / 0	1 / 6.7		
	cefazolin / gentamicin	2 / 9.1	1 / 16.7	1 / 6.7		
	clindamycin	0 / 0	0 / 0	2 / 13.3		
	ampicillin / sulbactam	2 / 9.1	0 / 0	0 / 0		
duration of initial antibiotic therapy	< 24 h	2 / 14.3	0 / 0	1 / 10.0	2.857 / 4	0.643
	24–72 h	4 / 28.6	4 / 66.7	4 / 40.0		
	> 72 h	8 / 57.1	2 / 33.3	5 / 50.0		
metal in situ	no	1 / 4.5	0 / 0	0 / 0	0.977 / 2	0.613
	yes	21 / 95.5	6 / 100	14 / 100		

### Microbiological patterns

Analyzing the microbial pattern, it became evident that *Stapylocooccus* spp. were most often detected by microbiological analysis (53.2%). Methicillin sensitive *Staphylococcus aureus* was in 26.1% the evidenced infection causing agent. Hereafter, *Enterococcus* spp. (22.5%), gram-negative bacteria (10.8%) and *Streptococcus* spp. (6.3%) were detected. *Streptococcus* spp. and gram-negative bacteria were only evidenced in cases with initial polymicriobial infection or germ-change in course of the treatment. *Enterococcus* spp. were more often detected in polymicrobial cases and germ-change in course of surgical revisions (Table 4). In twelve patients, sonication of metallic implants has been performed since 2016. In three patients the sonication led to the additional detection of bacteria, which otherwise could not be detected.

**Table 4:** *Streptococcu*s species and gram-negative bacteria were detected only in cases with polymicrobial infection and germ changes in course of the treatment. *Enterococcus* species were also detected more often in such cases, however not statistically significant

	monomicrobial		polymicrobial		germ-change	
	number [n]	percent [%]	number [n]	percent [%]	number [n]	percent [%]
*Staphylococcus aureus*	10	45.5	5	21.7	14	21.2
*Staphylococcus epidermidis*	5	22.7	3	13.0	10	15.2
*Staph. coagulase-negative*	2	9.1	1	4.3	4	6.1
*Staphylococcus capitis*	1	4.5	1	4.3	1	1.5
*Staphylococcus hominis*	1	4.5	–	4.3	1	1.5
MRSA	1	4.5	–	–	4	6.1
***Streptococcus*** **species**	**–**	**–**	**3**	**13.0**	**4**	**6.1**
***Enterococcus*** **species**	**1**	**4.5**	**4**	**17.4**	**20**	**30.3**
**Gram-negative bacteria**	**–**	**–**	**5**	**21.7**	**7**	**10.6**
others	1	4.5	1	4.3	1	1.5
total	22	100.0	23	100.0	66	100.0

## Discussion

In the present study 14.3% of the infected nonunions could be diagnosed as polymicrobial infection. Infected nonunions which were defined by FRI criteria and cessation of bone healing after at least six months can be regarded as chronic FRIs. Thus, infected nonunions can be compared to posttraumatic osteomyelitis being a chronic bone infection as well. Jorge et al. reported 37.8% polymicrobial infections using standard culturing techniques in posttraumatic osteomyelitis [12]. Wimmer and co-workers reported 46.6% polymicrobial infections in PJI [8], while Bozhkova et al. found 28.5% PJIs to be polymicrobial [13]. Both studies used standard culturing techniques and did not differ between acute and chronic PJI as well. In addition, comparing different entities such as PJI with infected nonunion makes comparison of data difficult. Reasons for the low rate of polymicrobial infections (Table 1) could be previous antibiotic treatment as part of fracture care. Preceding antibiotic prophylaxis in fracture treatment might reduce numbers of different germs at the fracture site and thus later polymicrobial infection in infected nonunion. Results, however, did not show any statistically significant difference of monomicrobial, polymicrobial infections as well as germ-changes in course of treatment in regard to type and duration of antibiotic treatment (Table 3), which might be due to the low volume in case numbers. Type of accident was considered an indicator of associated soft tissue damage. Data, however did not show any difference concerning type of infection. Open fractures are expected to be exposed to several different pathogens compared to closed fracture. Further, patients with open fractures usually obtain longer perioperative antibiotic treatment than patients with closed fractures in which antibiotic treatment is not recommended beyond 24 h [14]. Intriguinly, we found same numbers of open and closed fractures for polymicrobial infections in infected nonunion (*n* = 3 each). Since almost equal numbers of infected nonunions for closed (57.1%) and open fractures (42.9%) could be figured out, longer antibiotic treatment could be reason for similar, but not higher numbers of polymicrobial infections in open fractures compared to closed ones. Nevertheless, data suffices not to proof this theory. Another aspect which might explain low numbers of polymicrobial infection is tissue culture itself. It is traditionally the gold standard for diagnosing infection, although low sensitivity is reported for culturing intraoperatively taken tissue samples [15]. Hospital-based culture techniques apply heavy selection pressure. Different germs in originally polymicrobial infections could be simply overgrown in conventional plating. In addition, artificial growth conditions favor bacteria capable growing under those conditions [16]. Furthermore, biofilm bacteria which are in their sessile phase are generally difficult to culture [17]. Another aspect is the possibility of presence of viable but non-culturable bacteria (VBNC). Many planktonic as well as biofilm bacteria are able to enter a starvation survival state, which results from external stress (e.g. antibiotic treatment). Those bacteria can not be grown on laboratory media and are therefore difficult to detect with standard microbiological assays [18, 19]. Furthermore, additional PCR was not performed routinely in patients with positive culture results. Thus, the authors could not determine a significantly higher rate of polymicrobial infections as reported by Palmer and co-workers who compared PCR combined with time-of-flight mass spectrometer and Fluorescence In Situ Hybridization (FISH) with microbiological culture [15].

In literature, coagulase-negative *Staphylococcus* spp. and *Staphylococcus aureus* are the most common infection causing germs in infected nonunions and PJI [20–22]. We found *Staphylococcus* spp. being the most frequently evidenced pathogen in infected nonunion patients, followed by *Enterococcus* spp.. Interestingly, gram-negative bacteria and *Streptococcus* spp. were only found in polymicrobial infections and cases with germ-change evidenced after follow-up revision surgeries (Table 4). An explanation for detecting gram-negative bacteria in those cases might be empirical broad-spectrum antibiotic therapy with amoxicillin/clavulanic acid and generation I and II cephalosporines after the initial revision surgery. Those antibiotics target predominantly gram-positive pathogens. Since cefazolin was most often used for empirical treatment in infected nonunion treatment, resistance of *Enterococcus* spp. to cephalosporins may explain the more frequent detection of *Enterococcus* spp. after revision surgeries [23]. Despite the shift of detected germs during multiple revision surgeries, the present data shows that all germ-changes in course of multiple revision surgeries occurred in patients with initial monomicrobial infection (21/21 patients, 100%). This can either be attributed to superinfection during surgical treatment itself or to failure in microbiological detection of all pathogens responsible for index infection. Moreover, germ-changes may also be regarded as laboratory contaminations, especially towards the identification of coagulase-negative staphylococci. Even a standardiszd collection of relevant samples from the suspected osseous defect cannot exclude subsequent contamination within the framework of microbiological diagnostics [24].

Limitations of our study are its retrospective design and the low volume in numbers of patients. Since infected nonunion is a relative rare diagnosed sequela, not more than 42 patients were involved according to our inclusion criteria within a long observation period. The low number in cases with a small group of polymicrobial infections prevents statistically significant conclusion of the results. Inclusion of infected long bone nonunions of the lower and upper extremities leads to further heterogenity of the study group. Although different microbiological patterns in musculoskeletal infections of the upper and lower extremities are well known [25], the authors considered reporting those cases useful due to lack of reported microbial findings in infected nounions of the upper extremities. The retrospective design of the study accounts for a high rate of unknown previous antibiotic treatment. Patients suffering from infected nonunion usually undergo long-term suppressive antibiotic therapy. This previous treatment may alter the diagnostic yield significantly as well as microbiological patterns detected by different diagnostic methods. Retrospectively reviewing medical records over a ten-year period did not allow to discriminate between diagnostic yield of different diagnostic methods. This can be regarded as a major drawback of our study, although standardization of tissue sampling and diagnostic work-up was established for the included patients. The fact, that instead of five relevant tissue samples, at least three relevant tissue samples were sent to microbiological diagnostics is a further downside. The recent understanding, that five relevant tissue samples increase microbial sensitivity in PJI and FRI, was not known for the observation period and thus retrospective analysis not possible [26] . The same applies to the laboratory culture work-up. Recent findings of higher sensitivity when culturing tissue samples in liquid media was not established as microbiological culture standard in our laboratory over the whole study period. Nevertheless, the standard diagnostics in our study should be similar to most of the microbiological diagnostic set-ups performed worldwide which prompted us to report the present data. The additional use of sonication did only result in three additional evidenced pathogens in cases otherwise being treated as culture negative infected nonunion. These limited case numbers did not allow any conclusions for diagnostic relevance of sonication which needs to be clarified in future studies. In addition, an exact classification of soft tissue damage according to Gustilo-Anderson in open fractures was not possible reviewing patient records. Lastly, the question if adaption of antibiotic treatment to germ-changes evidenced after follow-up revision surgeries is appropriate for treatment cannot be answered by this descriptive analysis.

## Conclusion

A low rate of polymicrobial infections in infected nonunion was evidenced in the present study. Reason for low numbers of polymicrobial infections may be insufficient diagnostics in clinical routine. Prospective studies are necessary to verify low rates of polymicrobial infections using more sensitive diagnostic tools. Thus, relevance of microbiological testing results for antibiotic and surgical treatment in infected nonunion could be derived in future.

## Data Availability

The datasets used and/or analysed during the current study are available from the corresponding author on reasonable request.

## Abbreviations

AB: Antibiotics
ASA: American Society of Anesthesiologists
BHI: Brain heart infusion
BMI: Body mass index
FISH: Fluorescence In Situ Hybridization
PCR: Polymerase chain reaction
PJI: Periprosthetic joint infection
PMMA: Polymethylmethacrylate
MRSA: Methicillin resistant *Staphylococcus aureus*
VBNC: Viable but non-culturable
